# Correlates of unsuccessful smoking cessation among adults in Bangladesh

**DOI:** 10.1016/j.pmedr.2017.08.007

**Published:** 2017-09-06

**Authors:** Shariful Hakim, Muhammad Abdul Baker Chowdhury, Md Jamal Uddin

**Affiliations:** a Department of Statistics, Shahjalal University of Science & Technology, Sylhet 3114, Bangladesh; b Department of Emergency Medicine, University of Florida College of Medicine, Gainesville, 32610, FL, USA

**Keywords:** GATS, Smoking, Cessation, Tobacco, Bangladesh

## Abstract

Having 21.9 million adult smokers, Bangladesh ranks among the top ten heaviest smoking countries in the world. Correlates of unsuccessful smoking cessation remain unknown. We aimed to identify the correlates of unsuccessful smoking cessation among adults in Bangladesh.

We used data from the 2009 Global Adult Tobacco Survey (GATS) for Bangladesh. We compared socio-demographic, belief about health effect of smoking, and environmental characteristics of current smokers who had a recent failed quit attempt during the past 12 months of the survey (unsuccessful quitters) with those former smokers who had quit ≥ 12 months earlier of the survey and had not relapsed (successful quitters). Data were analyzed using logistic regression model and generalized estimating equations.

A total of 1552 smokers (1058 unsuccessful quitters and 494 successful quitters) aged 15 years and older who participated in the survey was included in this study. Among the smokers, 1058 (68%) were unsuccessful quitters. Our analysis showed that older aged, female, and higher educated smokers were less likely to quit unsuccessfully. Moreover, who believed that smoking causes serious illness were also less likely to quit unsuccessfully. For the interaction between place of residence and smoking rules inside home, we found that among the smoker's, in those house smoking was allowed, and who lived in urban place were less likely to be unsuccessful in quitting than those who lived in rural place.

Our findings suggest a cessation program that requires integrated approach with a view to considering these findings in setting up.

## Highlights

• The interaction effects for smoking cessation has been rarely investigated.
• Almost 70% of adult smokers were unsuccessful quitters.
• Older aged, female, and educated smokers were less likely to quit unsuccessfully.
• Belief about harmful effect of smoking associated with unsuccessful smoking cessation
• Interaction between smoking rules inside home and place of residence was observed.

## 1 Introduction

Worldwide, tobacco use is the leading cause of avoidable death (Abdullah et al., 2015). In each year, nearly 6 million peoples are killed by tobacco (World Health Organization, 2017c) and if present pattern of tobacco use remains uncontrolled, > 8 million deaths will be caused annually by 2030 (Jha, 2011). However, rates of smoking in developed countries is decreasing but in developing countries, it is rising (Giovino et al., 2012). The fast increase in smoking in developing countries would result in 7 million deaths per year by 2030 (Abdullah and Husten, 2004). The countries in Asia, especially, South East Asia are not unsusceptible to smoking epidemic (Rao et al., 2014). The South East Asia region is the place of residence for about 400 million tobacco users, which bring about 1.2 million deaths annually (Sreeramareddy et al., 2014).

Bangladesh is larger than most other tobacco consuming countries in the world where 46 million adults use tobacco (Barkat et al., 2012). Bangladesh ranks among the top ten heaviest smoking countries in the world having high current smoking prevalence of 44.7% among males, 1.5% among females, and overall 23.0% among adults aged 15 years or above (Nargis et al., 2015). This means an estimated 21.9 million adults in Bangladesh currently smoke tobacco (World Health Organization, 2017a). Bangladesh is one of the fifteen countries in the world having a greater burden of tobacco-attributable illness (World Health Organization, 2017b). An earlier study conducted by World Health Organization (WHO) in 2004, showed that tobacco causes approximately 57,000 deaths and 1.2 million tobacco-related illness in Bangladesh (World Health Organization, 2017a). Another study conducted in 2010, found that smoking was responsible for 25% of all deaths among Bangladeshi men aged 25 to 69 years and reduces their life expectancy by average 7 years (Alam et al., 2013). Moreover, because of tobacco-attributable deaths in Bangladesh, the health and economic burden are rising rapidly (Nargis et al., 2015). Therefore, to tackle this epidemic reducing commencement of tobacco use and widespread cessation can have a substantial effect.

Several studies that identify the factors that are associated with successful quit attempts have been restricted to specific populations such as young adults/adolescents (Chen et al., 2001, Rose et al., 1996, Tucker et al., 2005) clinic and/or patients (Foulds et al., 2006, Gelenberg et al., 2008), prisoners (Indig et al., 2013, Makris et al., 2012, Richmond et al., 2013). As far, we know, there are a few studies that have identified the correlates of successful smoking cessation in general populations (Jampaklay et al., 2015, Kaleta et al., 2014, Lee and Kahende, 2007, Kim, 2014).

In a recent study in Bangladesh, successful smoking cessation was associated with older age, perceiving good/excellent self-rated health a, and an increased level of self-efficacy (Abdullah et al., 2015). In a study done in Korean population, successful quitters were more likely to be aged 65 years or older, women, married, having higher education, having higher income, having a lower level of stress, having smoked 20 or more cigarettes per day, and one's will for quitting (Kim, 2014). In another study in the U.S. population, successful quit attempts were associated with smoke free-homes and no-smoking policy at work, older age (35 years or more), having at least a college education, being married or living with a partner, being a non-Hispanic White, having a single life time quit attempt, and not switching to light cigarettes (Lee and Kahende, 2007).

However, almost none of these studies have considered interaction effects between potential factors on the outcome variable in multivariable modeling where the effect of one factor may be different depending on the another factor (Afshartous and Preston, 2011). For example, the effect of smoking rules inside the smoker's home on the probability of successful smoking cessation may greater for who lives in urban areas than who lives in rural areas. Moreover, multi-stage sampling is used by almost all national surveys. Consequently, the collected data are clustered with a nested structure. One vital result of clustering is that measurement on units within a same cluster are correlated. To our knowledge, almost none of the studies that identified the characteristics of successful quitters did not consider clustering (if any) in the data set. Ignoring clustering effects in the data set may draw an invalid conclusion such as overestimating the variability, falsely increasing the p-values, reducing the statistical power, and increasing the chance of type-II error (Sainani, 2010).

To our knowledge, few studies specify the amount of smokers who have attempted to quit but failed (unsuccessful) and describe their characteristics. However, quitting smoking is a dynamic process and several unsuccessful quit attempts may be involved before finally succeeding (Larabie, 2005). Though many smokers are attempted to quit smoking but unsuccessful, it is important to take into consider their all quit attempts (Derby et al., 1994). Moreover, these unsuccessful quitters are at the minimum attempted to stop smoking underscores that they are intended, but because of their tobacco addiction, they are impotent to sustain continual abstinence (Lee and Kahende, 2007). Therefore, with a view to addressing all the impediments sufficiently to smoking cessation among these unsuccessful quitters, it is essential to identify the characteristics of the smokers who have tried to quit but unsuccessful. This study uses a large representative sample from a cross-sectional national survey in Bangladesh to determine the factors that are associated with the smokers who have unsuccessfully attempted to quit smoking during the past 12 months of the survey.

## 2 Methods

### 2.1 Data source and study population

We used latest nationally representative data from the 2009 Global Adult Tobacco Survey (GATS), Bangladesh (Centers for Disease Control and Prevention, 2017). The GATS, a component of Global Tobacco Surveillance System (GTSS), is a global standard for systematically observing adult (15 years of age or older) tobacco use and tracking key tobacco control measures. In Bangladesh, GATS was conducted in 2009 executed by the National Institute of Preventive and Social Medicine (NIPSOM) with the cooperation of National Institute of Population Research and Training (NIPORT), and the Bangladesh Bureau of Statistics (BBS). Centers for Disease Control and Prevention (CDC) and the WHO provided the technical support.

### 2.2 Sampling frame and samples design

**Fig. 1:**
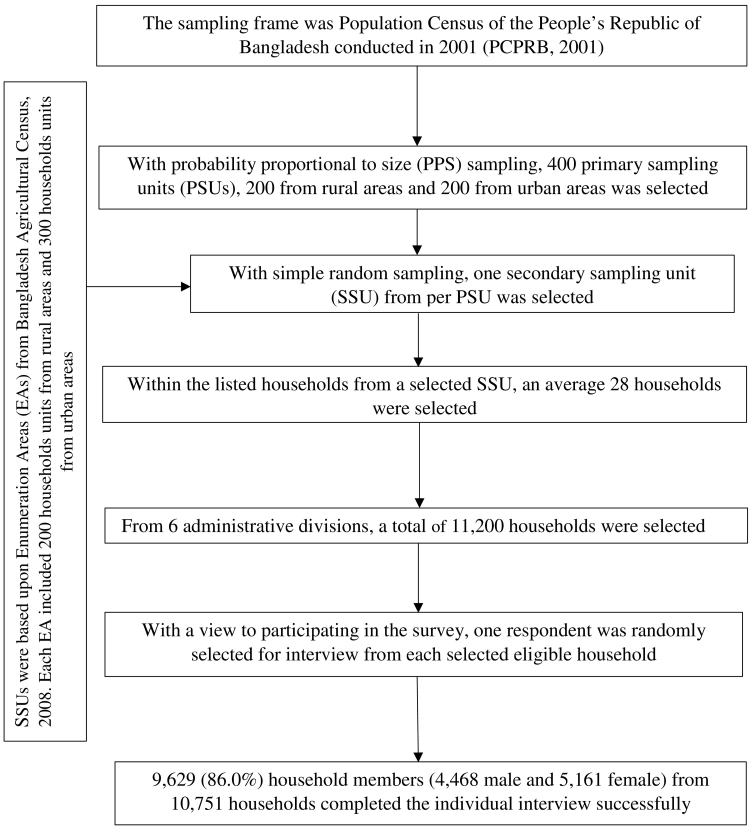
Study design of 2009 Global Adult Tobacco Survey Bangladesh.

The sampling frame for the 2009 GATS was used from the 2001 Population and Housing Census. The primary-sampling units (PSUs) were mahalla (for the urban stratum) and mauza (for rural stratum) and a three-stage stratified cluster sampling was used to draw sample. In the first stage, 400 PSUs were selected using probability proportional to size (PPS) sampling. In the second stage of sampling, one secondary sampling units (SSU) was selected from per PSU with simple random sampling (SRS). In the third stage, a systematic sample of 28 households on average from each SSU was selected to produce equal male and female households on design specifications (World Health Organization, 2009). With this design, the survey selected 11,200 households. Among the selected households, 10,050 were found to be an acceptable person for the single interview. Out of 10,050 households, 9629 individuals completed the interview successfully. The sampling procedure and the study design is presented in Fig. 1. The detailed survey procedure, study method, questionnaires are available in elsewhere (World Health Organization, 2009).

## 3 Measures

### 3.1 Outcome variable

**Fig. 2:**
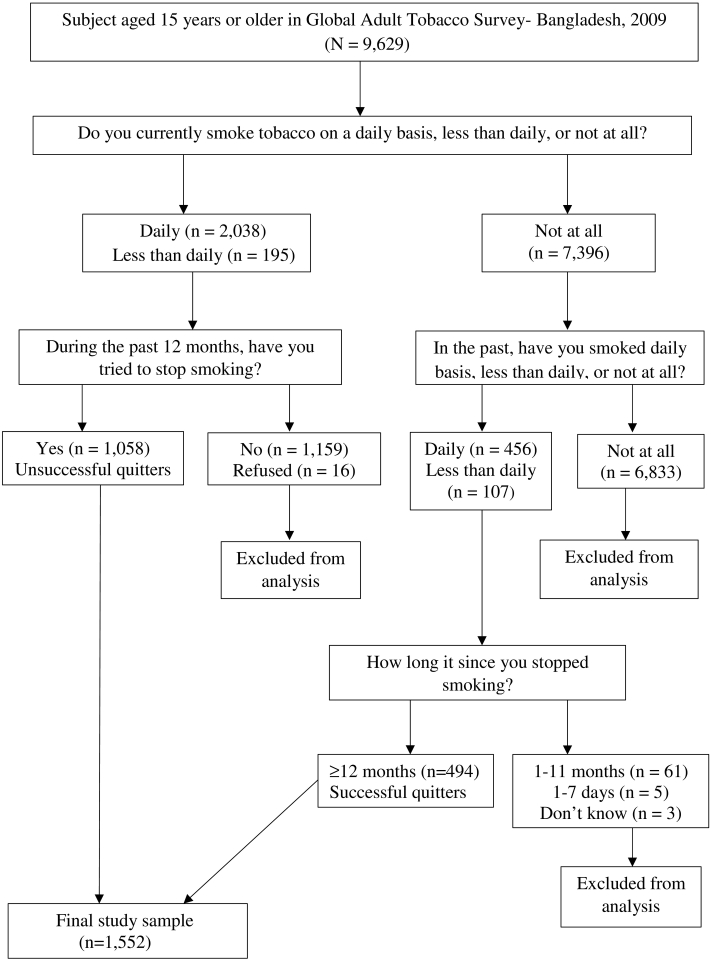
Survey screening process used to select current smoker with a recent failed quit attempt (unsuccessful quitters) and recent successful quitters.

We compared unsuccessful quitters with recent former smokers who had stopped smoking 12 months earlier of the survey and had not relapsed. The unsuccessful quitters were defined as those who reported that they smoke currently on daily basis or less then daily basis, and had tried to stop smoking, but recently failed (during the past 12 months of the survey). The successful quitters were defined as those who reported that they do not smoke currently, but they have smoked daily basis or less then daily basis in the past and have stopped smoking for > 12 months of the survey. The screening process used to select unsuccessful quitters and successful quitters is illustrated in Fig. 2.

### 3.2 Potential factors

Six socio-demographic characteristics such as age (categorized as 55 years or older, 45–54, 35–44, 25–34, and 15–24), gender (male, female), place of residence (urban, rural), occupation (employed, business, farmers, laborers, student, homemaker, and unemployed), education (secondary school and above completed, less than secondary school completed, primary school completed, less than primary school completed, and no formal education), wealth index (highest, high, middle, low, and lowest) was used in this study. Wealth index was created using principal component analysis (World Health Organization, 2009).

Beliefs about the health effects of smoking indicated, believe that smoking causes serious illness (yes, no), and believe that cigarettes are addictive (yes, no). Environmental characteristic indicated smoking rules inside the home (allowed, not allowed, but exceptions, never allowed, and no rules).

### 3.3 Data analysis

We compared the proportion of successful quitters and unsuccessful quitters between the categories of various independent variables. Binary logistic regression analysis and generalized estimating equations (GEE) with considering clustering effect in the data were used to identify the factors that are associated with unsuccessful smoking cessation. We included all potential factors in the multivariable full model. We evaluated multicollinearity using variance inflation factor (VIF) with a cutoff 4.0 (Pan and Jackson, 2008).

### 3.4 Variable selection and model diagnostics

We formed logistic regression model using backward elimination procedure. First, a full model was formed with all main effect and selected two-way meaningful interactions between factors (Hilbe, 2009). Then, at a time the term that has the highest p-value was eliminated from the model. The procedure was repeated until no (additional) effects met the 5% significance level for elimination from the model. Akaike Information Criteria (AIC) was calculated in each step. We select the final model based on the minimum AIC. With a view to assessing the overall fit of the final model, we used Pearson Chi-square and Hosmer-Lemeshow goodness of fit statistic (Hosmer et al., 2013). We did not find any lack of fit of the model (Table S1). In addition, to detect influential observation, Pearson residual and deviance residual were used. We used area under the curve (AUC) of receiver operating characteristic curve (ROC) to check the predictive accuracy of the final model (Fig. S2).

The GATS, Bangladesh-2009 data set used in this study was based on multistage cluster sampling. For this reason, the hierarchical structure of the data (Fig. S1) creates the dependence among observations. Hence, observations within a same cluster are correlated. With a view to taking into consideration the clustering effect in the data, we considered GEE (Hardin and Hilbe, 2012), which accounts the correlation among the observations within a cluster. To choose a working correlation structure, we used two methods GEE (Hardin and Hilbe, 2012). First, we choose a correlation structure that minimizes Quasi-Information Criteria (QIC), second, a correlation structure for which the empirical estimates of the variance most closely approximate the model-based estimate of the variance. Similar to the logistic regression model, the final GEE model was also selected using a backward elimination procedure. In each step, we computed QICu and the final model was selected based on the minimum QICu value (Pan, 2001). The number of covariates with interactions in logistic regression final model does not differ much from GEE final model. In GEE, we found only one additional main effect (occupation) and an interaction effect (place of residence*wealth) than logistic regression model.

Statistical software SPSS (version 21.0) and SAS version 9.4 (SAS Institute Inc., Cary, NC) were used for data management and analysis.

## 4 Results

### 4.1 Study subjects

Among the 9629 respondents aged 15 years or older who completed the survey, 2233 were current smokers, 563 were former smokers, and 6833 were never smokers. Among current smokers 1058 were unsuccessful quitters (who had tried to stop smoking but failed during the past 12 months of the survey). Of the former smokers 494 had quit 12 months earlier of the survey (successful quitters). The 69 former smokers were not included as a successful quitter in the analysis because they had quit 1 to 12 months earlier of the survey and they are probable to have had relapse (Lee and Kahende, 2007). Thus, 1058 unsuccessful quitters and 494 successful quitters were the final study subjects (Fig. 2).

### 4.2 Bivariate analysis

Table 1 shows the proportion of successful quitters and unsuccessful quitters between the categories of various potential factors. Among the male smokers, 69.8% were unsuccessful quitters, while among female this proportion was 36.5%, similar proportions of unsuccessful quitters were observed in rural and urban areas (68.1% vs. 68.2%). The highest proportion (86%) of unsuccessful quitters was observed among younger adults (age 25–34 years). Individuals who had less than primary education (72%) and belong to lowest wealth quintile (73.3%) had a higher rate of unsuccessful quitting smoking. Among different occupation groups laborers had the highest proportion of unsuccessful quitting (76.3%) followed by employed (71.7%) and business professionals (71.3%). For the belief about health effects of smoking variables, respondents those did not believe that smoking causes serious illness and those did not believe that cigarettes are addictive, 90.3% and 73% are unsuccessful quitters respectively. For the environmental characteristic, among the smokers house where smoking was allowed, 83.4% were unsuccessful quitters and among the smokers house where smoking was never allowed, 54.2% were unsuccessful quitters.

**Table 1:** Comparison of the distribution of successful and unsuccessful quitters across the categories of the potential factors.

Variables	Successful quitters		Unsuccessful quitters	
	n	%	n	%
Socio-demographic characteristics				
Age (yr)				
55 and above	239	60.8	154	39.2
45–54	101	34.0	196	66.0
35–44	82	21.1	306	78.9
25–34	48	14.0	295	86.0
15–24	24	18.3	107	81.7
Gender				
Male	447	30.2	1031	69.8
Female	47	63.5	27	36.5
Place of residence				
Urban	256	31.9	547	68.1
Rural	238	31.8	511	68.2
Wealth index				
Highest	116	41.3	165	58.7
High	99	30.7	223	69.3
Middle	87	31.5	189	68.5
Lowest	97	26.7	266	73.3
Low	95	30.6	215	69.4
Level of education				
Secondary and above	95	40.3	141	59.7
Less than secondary	75	29.6	178	70.4
Primary	51	35.2	94	64.8
Less than primary	71	28.0	183	72.0
No formal education	202	30.4	462	69.6
Occupation				
Employed	64	28.3	162	71.7
Business	92	28.7	229	71.3
Farmers	95	31.7	205	68.3
Laborers	123	23.7	396	76.3
Student	4	50.0	4	50.0
Homemaker	34	69.4	15	30.6
Unemployed	81	63.3	47	36.7
Missing	1	100		

Belief about health effect of smoking				
Believe that smoking causes serious illness				
Yes	489	32.4	1021	67.6
No	3	9.7	28	90.3
Missing	2	18.2	9	81.8
Believe that cigarettes are addictive				
Yes	456	32.2	960	67.8
No	33	27.0	89	73.0
Missing	5	35.7	9	64.3

Environmental characteristic				
Smoking rules inside home				
Allowed	58	16.6	291	83.4
Not allowed, but exceptions	98	32.9	200	67.1
Never allowed	201	45.8	238	54.2
No rules	137	29.4	329	70.6

### 4.3 Multivariable analysis

The results of the logistic regression model for unsuccessful smoking cessation are shown in Table 2. With respect to socio-demographic characteristics, the odds of unsuccessful smoking cessation decreased with age. Males were 6.18 times more likely (OR = 6.18, 95% CI: 3.43–11.14) to be unsuccessful in quitting smoking than female. People with secondary school or higher educational attainment were 0.57 times less likely (OR = 0.57, 95% CI: 0.39–89) to quit unsuccessfully than those with no formal education. With respect to belief about health effect of smoking, people who believed that smoking causes serious illness were 0.14 times less likely (OR = 0.14, 95% CI: 0.04–0.53) to quit unsuccessfully compared to who did not believe that smoking causes serious illness. For the interaction between place of residence and smoking rules inside home, we found that among the smoker's, in those house there were no rules about smoking, and who lived in urban place were 1.61 times more likely (OR = 1.61, 95% CI: 1.02–2.53) to be unsuccessful quitters than those who lived in rural place.

**Table 2:** Correlates of unsuccessful smoking cessation: odds ratio and 95% confidence intervals from multivariable logistic regression model.

Characteristics	Odds ratio	95% confidence limits	
		Upper	Lower
Age			
55 +	0.11	0.07	0.19
45–54	0.34	0.20	0.59
35–44	0.68	0.40	1.17
25–34	1.20	0.68	2.11
15–24®	1		
Gender			
Male	6.18	3.43	11.14
Female®	1		
Level of education			
Secondary school and above	0.57	0.39	0.84
Less than secondary school	0.73	0.51	1.07
Primary school	0.53	0.34	0.83
Less than primary school	0.81	0.56	1.18
No formal education®	1		
Place of residence			
Urban	–	–	–
Rural®			
Believe that smoking causes serious illness			
Yes	0.14	0.04	0.53
No	1		
Smoking rules inside home			
Allowed	–	–	–
Not allowed, but exceptions	–	–	–
Never allowed	–	–	–
No rules®			
Place of residence ∗ smoking restrictions inside home			
Urban vs. rural at allowed	0.93	0.49	1.77
Urban vs. rural at not allowed, but exceptions	1.08	0.63	1.85
Urban vs. rural at never allowed	0.61	0.39	0.94
Urban vs. rural at no rules	1.61	1.02	2.53

Note: ® = Reference category

## 5 Discussion

We found the correlates of unsuccessful smoking cessation were age, gender, level of education, place of residence, believe that smoking causes serious illness, smoking rules inside home. We also found a significant interaction between place of residence and smoking rules inside home. Moreover, we found approximately similar results from both analyses ignoring and considering clustering effects in the data. This finding indicates that the clustering effect in the data may not be notable.

Consistent with findings from previous research (Lee and Kahende, 2007, Hymowitz et al., 1997, Kim, 2014), in this study, we found that young adult smokers (25 to 34 years) have higher unsuccessful quit rate compared with older adults. The probable explanation of this association is that young adults faced less health problems, which do not cause the risk of smoking apparently. On the other hand, older smokers make multiple visit to health care providers and receive advice from them to quit which influence them to succeed in quitting smoking (Abdullah et al., 2015). Moreover, it is also investigated that older smokers are more likely to show manifestation of smoking-attributable illness, which also may strengthen their intention to quit (Kaleta et al., 2014). Thus, our findings suggest that it is necessary to promote targeted smoking cessation interventions for young adults in order to quit smoking successfully.

There is conflicting result of gender for predicting smoking cessation. Some studies (Hyland et al., 2004, Hymowitz et al., 1997) found that male smokers were more likely to be successful quitters and other studies (Chen et al., 2001, Derby et al., 1994, Rose et al., 1996) found no association between gender and successful quitting from smoking. Surprisingly, we found that female smokers were less likely to be unsuccessful quitters than male smokers, which is consistent with the findings from (Tillgren et al., 1996, Waldron, 1991, Kim, 2014). In the present study, among unsuccessful quitters there were only 2.6% women and among successful quitters there were 9.5% women. In Bangladesh, unlike western societies but like other Asian societies, relatively few women smoke (Flora et al., 2009). However, the female smokers are aware about the harmful effect of smoking especially during pregnancy and childcare which may influence them to quit successfully from smoking (Kim, 2014). On the other hand, the male smokers may highly addict to smoking. In addition, they may think, they will quit permanently after experiencing several negative impact of smoking and for this reason, they are failed to succeed in quitting. Thus, this study suggests that to discourage men from smoking and encourage them about the importance of quitting, it is also necessary generating gender-specific research and programs on the prevention of smoking in men.

Consistent with the previous findings (Kim, 2014, Wetter et al., 2005, Koning and Webbink, 2010), we found that education is a potential predictor of smoking cessation. In our study, we found that, unsuccessful smoking cessation rate is decreased with the increase in the level of education. Now-a-days smoking is not so much common among highly educated people. In a study (Koning and Webbink, 2010), they found that, a higher level of education raise the odds of smoking cessation rather than reducing the smoking initiation and they also showed that the duration of smoking with 9 months is (Schaap et al., 2008) reduced due to one additional year of education. Another study of 18 European countries (Schaap et al., 2008), noted that smokers with lower education were less likely to have quit smoking than smokers with higher education in all countries. Factors that may influence variation in quit rates among the smokers with lower and higher educational attainment may comprise general health knowledge, attitude, and beliefs (Kaleta et al., 2012). With a view to discouraging unsuccessful smoking cessation among lower educated smokers, targeted policies and interventions should be focused.

Consistent with findings from previous research (Ayo-Yusuf and Szymanski, 2010, Tejada et al., 2013), in this study, who didn't believe that smoking causes serious illness were more likely to be unsuccessful in quitting. The smokers who are unconscious that smoking is menacing, or who do not believe that smoking causes serious illness, are not more likely to make quit attempt; and when they try to do so, they are more likely to be unsuccessful (Ayo-Yusuf and Szymanski, 2010). Furthermore, smokers may think that they will stop smoking after they experiencing adverse effect of smoking. Therefore, it is essential for tobacco control messages to highlight the importance of stopping smoking earlier rather than later (Waters et al., 2016).

In our study, we found a significant interaction between smoking rules inside home and place of residence. From the interaction, we found that, among the smokers house where smoking was never allowed, and who lived in urban areas were less likely to quit unsuccessfully than rural smokers. In addition, we found that, though smoking was allowed in urban smoker house, they were more likely to quit successfully. A study found that in Bangladeshi urban residents had significantly higher likelihood of having smoke-free homes compared to rural residents (Abdullah et al., 2011). Our findings suggest that increased public education campaign about the harmful effect of secondhand smoke and the benefits of quitting in both urban and rural areas may influence smokers to stop smoking in the home voluntarily (Ayo-Yusuf and Szymanski, 2010).

### 5.1 Strengths and limitations

Our study has several strengths: first, the present study with a nationally representative sample was distinctive in its comprisal of unsuccessful quitters as well as its inclusion of predictors with interactions. Second, we assessed our final model using several model diagnostic tools. Finally, our final statistical model has a good prediction power. There are some notable limitations of our study. First, an important variable “number of cigarettes smoked per day” were not available for successful quitters in the data set. Second our study is a cross sectional study. For this reason, we are not able to see the changes over time. Third, since no cross-sectional data have been released after the 2009 GATS for this country, we used this old data in the present study. As the data was collected about ten years ago, smoker attitudes and beliefs may be changed over a couple of years. Fourth, the definition of unsuccessful quitters and successful quitters was based on a single question of “Do you currently smoke tobacco on a daily basis, less than daily, or not at all?” Fifth, similar to a large number of population-based studies, the GATS depends on self-reported smoking status and cessation behaviors. Because of this smoking could be under-or over-reported. Sixth, a number of important factors such as self-efficacy, number of previous quit attempt, marital status, monthly income, and number of smokers in the household that may also have associated with smoking cessation were not included in this study as they were not available in the dataset. Seventh, quitting methods used by the former smokers were not available in the data set and age of smoking initiation, time to first smoking after waking up also were not available for successful quitters in the data set. Finally, as we only considered smokers of age 15 years and older, the findings may not be generalizable to the younger age groups.

## 6 Conclusions

The present study findings confirmed that age, gender, level of education, believe that smoking causes serious illness, place of residence, and smoking rules inside home contributed to unsuccessful smoking cessation among adults in Bangladesh. Moreover, we found that the effect of smoking rules inside home on unsuccessful cessation depends on the smoker place of residence. We recommend a targeted intervention plan for those smokers, particularly who lives in rural areas, younger age group and had no formal education and simultaneously, implementing tobacco control strategies and programs that assist smoking cessation in Bangladesh.

## Appendix A Supplementary data

Supplementary material Image 1
