# Supplementary material for: Correlates of unsuccessful smoking cessation among adults in Bangladesh
Source: Prev Med Rep. 2017 Sep 6;8:122–8. doi: 10.1016/j.pmedr.2017.08.007 (PMC5633848; doi:10.1016/j.pmedr.2017.08.007)

**SUPPLEMENTS**

T**able S1:** GOF statistics for model unsuccessful smoking cessation vs. successful smoking cessation

| **Criterion** | **Value** | **DF** | **P-value** |
| --- | --- | --- | --- |
| Pearson | 612.03 | 578 | 0.1583 |
| HHosmer-Lemeshow | 6.27 | 8 | 0.6171 |

**Figure S1:** Clustered structure: Global Adult Tobacco Survey-Bangladesh, 2009.


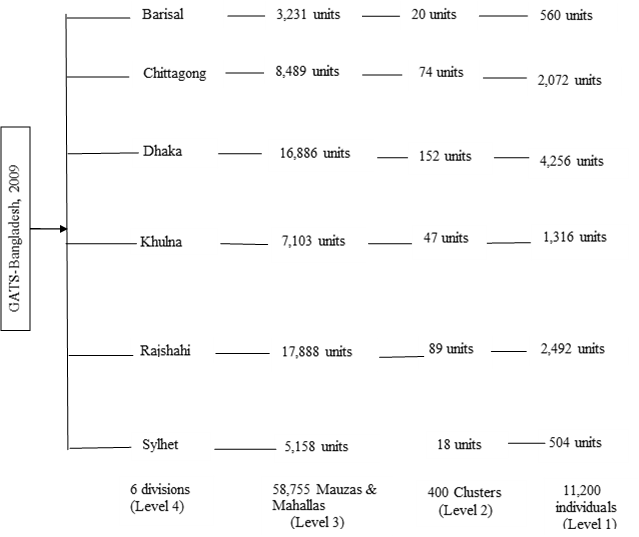


**Figure S2:** ROC curve for model unsuccessful smoking cessation vs. successful smoking cessation.


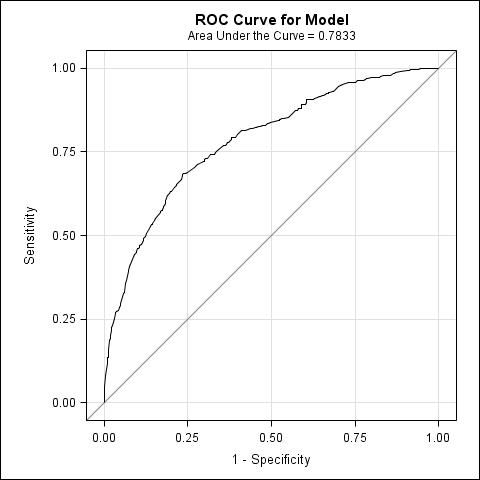

Supplement: Supplementary file 1 — Supplementary material [file mmc1.docx]
